# Its2vec: Fungal Species Identification Using Sequence Embedding and Random Forest Classification

**DOI:** 10.1155/2020/2468789

**Published:** 2020-05-27

**Authors:** Chao Wang, Ying Zhang, Shuguang Han

**Affiliations:** ^1^Institute of Fundamental and Frontier Sciences, University of Electronic Science and Technology of China, Chengdu 610054, China; ^2^Department of Pharmacy, Heilongjiang Province Land Reclamation Headquarters General Hospital, Harbin 150088, China; ^3^Center for Informational Biology, University of Electronic Science and Technology of China, Chengdu 60054, China

## Abstract

Fungi play essential roles in many ecological processes, and taxonomic classification is fundamental for microbial community characterization and vital for the study and preservation of fungal biodiversity. To cope with massive fungal barcode data, tools that can implement extensive volumes of barcode sequences, especially the internal transcribed spacer (ITS) region, are necessary. However, high variation in the ITS region and computational requirements for processing high-dimensional features remain challenging for existing predictors. In this study, we developed Its2vec, a bioinformatics tool for the classification of fungal ITS barcodes to the species level. An ITS database covering more than 25,000 species in a broad range of fungal taxa was assembled. For dimensionality reduction, a word embedding algorithm was used to represent an ITS sequence as a dense low-dimensional vector. A random forest-based classifier was built for species identification. Benchmarking results showed that our model achieved an accuracy comparable to that of several state-of-the-art predictors, and more importantly, it could implement large datasets and greatly reduce dimensionality. We expect the Its2vec model to be helpful for fungal species identification and, thus, for revealing microbial community structures and in deepening our understanding of their functional mechanisms.

## 1. Introduction

Metabarcoding is among the most promising approaches in the study of microbial communities [1–3] and has provided new insights into microbial impacts on crop yields [4], human health [5], and ecology [6]. Fungi are immensely diverse; the latest best estimate within this kingdom suggests that their total species number is somewhere between 2.2 and 2.8 million [7]. To date, only 144,000 (less than 7%) fungal species have been named and classified, while the vast majority are currently unknown to science [7]. Fungi play essential roles in many ecological processes as organic matter decomposers, mutualists with algae and plants [4, 8], plant pathogens, and components of the food chain [9–11]. Taxonomic classification is fundamental for microbial community characterization [12] and is vital for the study and preservation of fungal biodiversity [13]. However, it is difficult to identify specimens when their morphological characters are lacking or incomplete [14]. Several rRNA genes have been successfully employed for fungal species identification, including the small ribosomal subunit, the large ribosomal subunit, the RNA polymerase II binding protein, and the internal transcribed spacer (ITS). Among these, the ITS (including ITS1 and ITS2 separated by the 5.8S genic region) has been widely adopted as a marker for fungal identification and diversity exploration [15–19] because this region is ubiquitous and shows great variation in sequence and length [9].

Several ITS reference databases for fungal species identification have been developed. The UNITE database [20] and Warcup training set [9] are the most commonly used. The UNITE database (https://unite.ut.ee/) was first released in 2003 and focused on ectomycorrhizal fungi in north Europe [21], and it has been under successive development since then. UNITE aims to collect and disseminate all fungal ITS metadata from all geographical regions [20]. The latest version of the UNITE database (version 8.0) comprises approximately 1,000,000 public fungal ITS sequences (~459,000 species) for reference and provides valuable data for metabarcoding software pipelines [14]. The Warcup training set was developed from the UNITE database and includes only sequences with authoritative taxonomic or lineage information [9]. In addition, ITS barcodes in the BOLD database (http://www.boldsystems.org/) [22] and the ITS1 database comprising sequences of NCBI GenBank (http://www.ncbi.nlm.nih.gov/) have been used for fungal species identification [10, 15]. DNA barcode-based taxonomic assignment can be achieved by using similarity-based or prediction-based (alignment-free) methods. Similarity-based methods (e.g., BLAST) align the query sequence with all sequences in the reference database, which is time-consuming and inefficient when compared to alignment-free methods [1, 10]. Several prediction-based methods for fungal species prediction, including RDP classifier [9, 23], SINTAX [12], Mycofier [10], Mothur [24], and funbarRF [15], using various machine learning algorithms, have been developed in the past few years. To generate feature vectors, *k*-mer and its derivative, spaced *k*-mer, have been used for sequence encoding. The RDP classifier, which implements a naïve Bayes algorithm for taxonomy assignment, uses 8-mers as features [9]. SINTAX and Mothur, which use a non-Bayesian [12] and the *k*-nearest neighbor (kNN) algorithm [24], respectively, also use 8-mers. The naïve Bayes classifier Mycofier uses 5-mer features [10]. The random forest- (RF-) based predictor funbarRF uses spaced *k*-mer features for taxonomy classification [15].

Although great progress has been made in fungal species identification using machine learning algorithms, there still is room for further improvement. First, the species in the abovementioned datasets are only a small fraction of the species that have been named and classified. For example, the Warcup training set (version 2) covers 8,551 species of 1,461 genera [9], the ITS database of BOLD includes 3,674 species of 777 genera [15], and the ITS1 database of NCBI comprises 1,794 species of 510 genera [10]. A larger dataset that covers a broad range of fungal taxa would provide more valuable insights into microbial community compositions. Second, the *k*-mer-based representation method counts the frequency of all possible subsequences of length *k* of a sequence, which usually yields high-dimensional (i.e., 4^8^ for RDP classifier) and sparse vectors [25]. To address this issue, a representation method that can reduce dimensionality and encode each sequence into a dense, numeric vector is required.

Feature extraction is very important for constructing a computational predictor [4, 26–40]. Recently, a new efficient method for nucleotide sequence representation was proposed using a word embedding algorithm [41], such as word2vec [42]. Word embedding was originally developed for natural language processing [41]. In this model, each word is characterized by its context, i.e., neighboring words, and embedded in a predefined *n*-dimensional vector, where similar words have close vectors. This word representation method has been successfully employed to generate features from biological sequences. Asgari and Mofrad [43] applied the word2vec framework to represent and extract features of DNA sequences and protein families and achieved an average classification accuracy of 93% based on the classification of 7,027 protein families. Subsequently, a number of studies using this approach for the distributed representation of biological sequences were reported, including analyses of DNA [44–46], non-coding RNA [47], long non-coding RNA [48–50], and 16S rRNA [25].

The aim of this work was to develop a machine learning-based classifier for classifying fungal DNA barcodes. The filtered UNITE database covering broad range of fungal taxa was constructed, and a word embedding algorithm was employed to represent ITS sequences as dense, low-dimensional vectors. We demonstrate that this novel tool name “Its2vec” achieved an accuracy comparable to that of state-of-the-art predictors. We expect that Its2vec can aid in the computational classification of fungal species.

## 2. Materials and Methods

### 2.1. ITS Database and Preprocessing

One of our goals was to gain a deeper insight into microbial community structures by developing a database that contains as much representative fungal species as possible. The UNITE_public database contains fungal ITS sequences from both the International Nucleotide Sequence Database Collaboration (INSDC) and UNITE dataset [16, 51]; the latest UNITE_public (INSDC+UNITE) v8.0 includes 887,397 sequences. The dataset was clustered at several similarity thresholds to obtain species-level operational taxonomic units, referred to as species hypotheses (SHs); for each SH, if two or more ITS sequences are available, a representative sequence was chosen randomly to represent the SH [16], which resulted in the sh_general database, including 35,667 sequences (https://unite.ut.ee/repository.php) [20].

**Figure 1:**
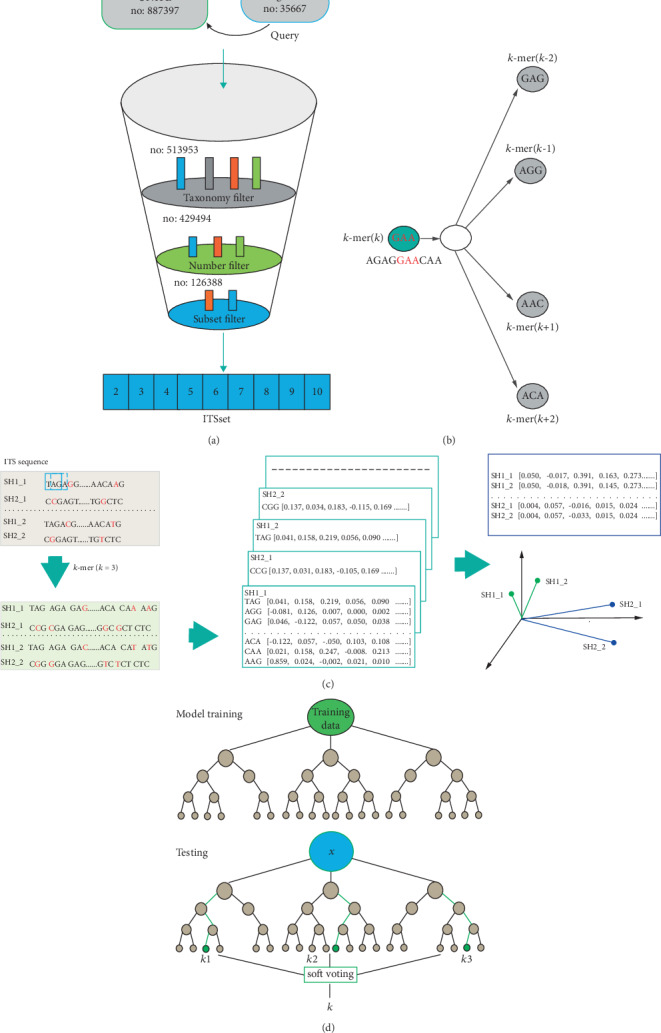
Schematic view of Its2vec. (a) Pipeline scheme of ITS dataset construction. (b) The skip-gram architecture of word2vec, which predicts surrounding *k*-mers (GAA, AGG, AAG, and ACA) based on a given center word (GAA). (c) Pipeline scheme of distributed representation of ITS sequences. For example, the ITS sequence SH1_1 (length *N*) was first represented by an *N*-2 3-mer set (TAG, AGA, GAG,…, AAG). Then, for each *k*-mer, we generated a distributed vector representation based on the skip-gram model with a vector of size 100, i.e., TAG [0.041, 0.158, 0.219…]. Thus, sequence SH1_1 was represented by the average of all *n*-2 *k*-mers, which also is a vector of size 100, i.e., SH1_1 [0.050, –0.017, 0.391…]. Similar words have close vectors; in this figure, SH1_1 and SH2_1 are close to SH1_2 and SH2_2, respectively. (d) Flow diagram showing model training and testing using the RF classifier.

In this study, an ITS database was developed starting from the sh_general database. Sequences assigned a SH in the UNITE_public were extracted using sequences in sh_general as a query, resulting in a dataset with 513,953 sequences belonging to 32,523 SHs (at least 2 representative sequences for each SH). Then, any sequences without clear taxonomic information at the genus and species levels (i.e., g__unidentified; s__uncultured) were discarded. Thus, 429,494 sequences confined to 27,520 SHs were obtained (Figure 1(a)). As some SHs were represented by hundreds of sequences, to reduce the heterogeneity in sequence numbers, 10 sequences were randomly selected for SH that contained more than 10 sequences. Finally, 126,388 sequences belonging to 27,520 SHs were retained for analysis.

### 2.2. Distributed Representation of ITS Sequences

A distributed representation of the ITS sequence was generated in two major steps. First, the ITS sequences were lexically represented as a large set of *k*-mers (Figure 1(c)). For a sequence of length *N*, *N* − *k* + 1 *k* − mers were generated by moving a window of size *k* along the sequence. These *k*-mers [52] are similar to the words belonging to a corpus in natural language processing. Ten datasets with *k*-mer lengths ranging from 3 to 12 were evaluated. Given this *k*-mer set, the second step was to train the distributed representation. *k*-mer embedding training was processed using the skip-gram model of word2vec [41, 53, 54] implemented in Gensim 3.40 (https://radimrehurek.com/gensim/apiref.html) (Figure 1(b)). Wet set min_count = 1, epochs = 5, and the window size was varied from 2 to 9; other parameters were set to their default value. Thus, each *k*-mer was presented as a numeric vector of size 100, and each sequence (length *k*) was represented by the average of all vectors of *N* − *k* + 1 *k* − mers, which also is a vector of size 100 (Figure 1(a)).

### 2.3. Classifier and Dataset for Training and Validation

Several supervised learning techniques, such as naïve Bayes [9, 10, 12, 55], kNN [24], and RF [15, 35, 56–61], have been used for predicting ITS sequences. In this study, RF was selected for the modeling of ITS sequences because it is a powerful machine-learning algorithm that is nonparametric, robust to noise, and suitable for large datasets [62] (Figure 1(d)). For each SH, the class label was assigned to an integer and the number of classes was equal to the number of SHs, namely, 25,720.

The filtered database contained more than 25,000 SHs, and each SH was represented by at least 2 sequences. Given the extensive dataset (including more than 120,000 sequences) and the heterogeneity in sequence numbers among species, training and validation on the whole dataset would be arduous. Therefore, the ITS dataset was divided into 9 subdatasets, termed ITSset_2 to ITSset_10. Each subdataset contained species represented by a specific number of sequences, i.e., ITSset_2 contained species with 2 representative sequences. Detailed information on sequences and species in each subdataset is presented in Table 1. For the ITSset with *k* (*k* ≥ 2) sequences per SH, *k*-fold cross validation (CV) was employed for model evaluation. The ITSset was split into *k* smaller subsets, and a model was first trained by *k*-1 subsets and then validated on the remaining subset. Note that the ITSser_9 contains 5,374 SHs (10 representative sequences per SH); thus, training on this subset on RF is challenging and therefore, 5 sequences were randomly selected for each SH of ITSset_9. Hence, for ITSset_9, the model was evaluated on 26,870 sequences of 5,374 SHs (5 representative sequences per SH).

We introduced 4 standard metrics, Accuracy, Recall, Precision and Mathew's correlation coefficient (MCC) [31, 50, 63–77], to evaluate the performance of the proposed models:

(1) $$\begin{array}{c} \text{Accuracy}=\frac{\text{TP}+\text{TN}}{\text{TP}+\text{TN}+\text{FP}+\text{FN}},\\ \text{Recall}=\frac{\text{TP}}{\text{TP}+\text{FN}},\\ \text{Precision}=\frac{\text{TP}}{\text{TP}+\text{FP}},\\ \text{MCC}=\frac{\text{TP}\times \text{TN}-\text{FP}\times \text{FN}}{\sqrt{\left(\text{TP}+\text{FP}\right)\left(\text{TP}+\text{FN}\right)\left(\text{TN}+\text{FP}\right)\left(\text{TN}+\text{FN}\right)}}, \end{array}$$

where TP is true positive, FP is false positive, FN is false negative, and TN is true negative.

### 2.4. Comparison with Other Fungal Classification Methods

The performance of the Its2vec model was compared with that of 3 other predictors, namely, RDP classifier, funbarRF, and Mothur. The executable source codes of RDP classifier (https://sourceforge.net/projects/rdp-classifier/), funbarRF (https://cran.r-project.org/web/packages/funbarRF/) and Mothur (https://github.com/mothur/mothur/releases/tag/v1.40.5) were downloaded to a local machine and applied to 2 fungal datasets. The performance of the 4 methods was first evaluated on the ITSset_5, which contained 1,684 SHs (5 sequences per SH). The training datasets Warcup of RDP and ITSset_5 were extracted from the UNITE database. Sequences in the datasets of funbarRF and the Warcup training set were approximately 97% nonredundant. Hence, the second dataset Fold-10, containing 1,084 species (10 sequences per species) after removing the 5.8S sequences and ITS1 sequences of the original dataset of funbarRF, was further used for model evaluation in this study as recommended by Meher et al. [15]. Accuracies were calculated over 5-fold CV for ITSset_5 and 10-fold CV for dataset Fold-10.

## 3. Results

### 3.1. Evaluation of Sequence Embedding on Fungal ITS

We first evaluated the performance of sequence embedding. All 126,388 sequences were represented by a *k*-mer corpus. Then, the *k*-mer embedding space was obtained by training a skip-gram model of word2vec on the corpus. Thus, each ITS sequence was represented as a numeric vector of size 100. In this process, two parameters, the length *k* of the *k*-mer and the window size *w* of the skip-gram model, were optimized. The accuracy of models constructed with different *k* is shown in Table 2. The classification accuracy improved by 1–3% when *k* ranged from 3 to 12 (Table 2). It can be seen that the accuracy was the highest when *k* was near to 9; i.e., the accuracy reached a maximum at 9-mer for 7 subsets and at 8-mer and 10-mer for the remaining 2 (Table 2). Subsets with a larger number of sequences per SH (species) yielded a higher accuracy, ranging from 68% for 2 sequences per SH to 97% for 9 sequences per SH. Similar results were obtained for the others 3 metrics, recall, precision, and MCC, where the maximum value was obtained at 9-mer for most subsets. Detailed results are provided in Supplementary Tables S1, S2, and S3. The optimum value of *k* was set as 9 in following experiments.

The window size *w* of the skip-gram model was varied from 1 to 7, and the classification accuracy was higher when *w* was near to 4 for datasets having a rather low number (2–4) of sequences per SH, whereas a higher accuracy was obtained at *w* = 2 for subsets containing more than 5 sequences per species (Table 3). For *w* larger than the above thresholds, the accuracy slightly decreased or stabilized. The accuracy score was 71.65% for ITSset_2 (2 sequences per SH), and it gradually increased with the number of representative sequences for each SH and reached 97.02% in ITSset_9 (9 sequences per SH) (Table 3). For other metrics (precision, recall, and MCC), findings were similar; detailed results are provided in Supplementary Tables S4, S5, and S6. Considering the improvement in the accuracy for SHs represented by low number of sequences, *w* was set to 4. As the 4 evaluation metrics showed similar variation tendencies in the 9 subsets, subsequent experiments were conducted using ITSset_5 and ITSset_7, for simplicity.

### 3.2. RF Classifier

**Figure 2:**
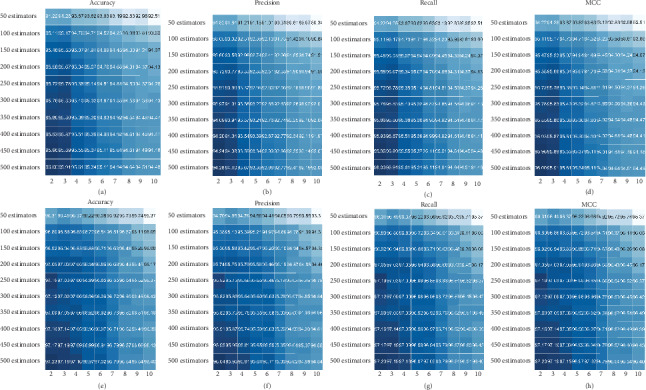
Accuracy, precision, recall, and MCC values of the RF model constructed using different numbers of features and estimators. (a–d) represent evaluation results of ITSset_5; (e–h) represent the evaluation results of ITSset_7.

Random forest (RF) was widely employed in the bioinformatics researches [78–81]. Two key parameters of the RF classifier were optimized, namely, the number of features considered for splitting at each leaf node (max_features) and the number of trees in the forest (*n*_estimators). By default, max_features and *n*_estimators were set to 10 (square root of features) and 100, respectively. We varied the two parameters to generate 90 models, where max_features was varied from 2 to10 in intervals of 1 and the *n*_estimators was varied from 50 to 500 in intervals of 50.  Figure 2 shows heat maps of accuracy, recall, MCC, and precision across the parameter combinations. In the accuracy heat map of ITSset_7 shown in Figure 2(e), it can be observed that the values gradually increase from the right upper region to the lower left region of the heat map, where the number of estimators linearly increased and the number of features decreased. Maximum values were obtained for the model based on 2 features and 500 estimators. The recall, MCC, and precision values are shown in Figures 2(f)–2(h); regions with more intense color in the three panels largely correspond to those in Figure 2(e). The evaluation results for ITSset_5 are shown in Figures 2(a)–2(d). It can be noted that higher values in the heat maps are observed in the similar regions of heat maps for ITSset_7. The values of the 4 metrics obtained for the model based on 2 features and 100 estimators were very close to the maximum values in the lower left region. Thus, considering the computational resources and the extensive dataset in this study, max_features was set to 2 and n_estimators was set to 100.

### 3.3. Performance Analysis Based on Optimized Parameters and Comparison with Other Predictors

The performance of the classifier was evaluated on all 9 subsets based on the optimized parameters (*k* = 9, window = 4, max_features = 2, and *n*_estimators = 100).  Table 4 shows the values of accuracy, precision, recall, and MCC for the datasets. The accuracy was 78.62% for ITSset2, which contains only 2 sequences per SH. With an increasing number of sequences in the SHs, the accuracy increased, and it reached a maximum value of 97.53% for ITSset_9. The precision, recall, and MCC showed similar trends, reaching maximum values of 96.37%, 97.53%, and 0.98 on ITSset_9, respectively.

The predictive power of Its2vec was compared with that of three state-of-the-art predictors, using two benchmark datasets. The results are presented in Table 5. The accuracy of Its2vec (95.51%), Mothur (97.80%), and RDP (98.68%) was significantly higher than that of funbarRF (91.0%) for dataset ITSset_5. For the Fold-10 dataset, Its2vec achieved a better performance than the other approaches; its accuracy was 0.54%, 4.26%, and 4.86% higher than that of RDP, Mothur, and funbarRF, respectively. Thus, Its2vec had an accuracy comparable to that of RDP and Mothur for ITSset_5 of the UNITE database and outperformed the other 3 predictors when applied to the Fold-10 dataset of BOLD.

## 4. Discussion

Fungi play essential roles in many ecological processes. Taxonomic classification is fundamental in functional investigations and endangered species conservation. The ITS region has been widely used as a DNA barcode for fungal species classification as it has a high PCR amplification success rate and species discriminatory power within the fungal kingdom [10]. Commonly used alignment-based methods often assign unidentified barcodes to species based on information on the cluster they are of in the barcode tree [82]. However, sequence alignment may be difficult for distantly related species due to the variability in nucleic acid base pairs and sequence length. Further, alignment-based methods are not suitable for metabarcoding analysis. In this study, we developed a new fungal ITS classification approach that uses a distributed representation technique to generate features of ITS sequences and applies RF for species identification.

Previously assembled fungal datasets are rather small; with 8,551 species of 1,461 genera, the Warcup training set currently is the largest. The latest version of the UNITE database (version 8.0) covers ~459,000 species and thus provides valuable data for metabarcoding software pipelines [20]. One of the main aims of this study was to develop an ITS database that covers a broad range of fungal taxa. After data filtering, 126,388 sequences belonging to 27,520 SHs (species)—which is three times the number in the Warcup training set—were retained for analysis. Generally, the sequence identities in a dataset are kept <80% to avoid overestimation. However, as the number of sequences for each SH is very small (2–9) and the numbers of classes are rather large (more than 250,000), this preprocessing step was not feasible in this study. To the best of our knowledge, none of the existing species identification studies using DNA barcodes [1, 9, 10, 12, 15, 24] have reported such a preprocessing step. When applied to large datasets, classifiers directly using *k*-mer-based features (commonly, 8-mer, 4^8^ features) are constrained by computational power. A word embedding algorithm was employed to represent each ITS barcode sequence as a dense, 100-dimensional vector.

To optimize the distributed representation of the ITS sequence, the length *k* of *k*-mer was optimized. We found that 8- and 9-mers resulted in the best performance. This implied that a *k* near 9 might be more informative, whereas a *k* larger than 10 may result in redundancy as evidenced by our results (Table 2). Similarly, RDP [9], SINTAX [12], and MOTHUR [24] use 8-mers. As for the window size of the skip-gram model, we noticed that a smaller window resulted in a higher accuracy, especially for SHs represented by more than 5 sequences (*w* = 2), which suggests that numbers of neighboring words predicted by the input (center) word are related to sequence abundance. Concerning RF classifiers, more trees always lead to a better performance and a more robust model, as shown in Figure 2, but can be associated with an excessively long training time and high computer memory demands. In our study, the computational capacity was exceeded when the model was applied to the ITSset_3 (12,423 sequences) with *n*_estimators = 450. Two parameters, max_features and *n*_estimators, were varied to determine the best split of the tree. Larger features will decrease the accuracy (Figure 2), indicating that a higher accuracy is obtained when the trees in the RF show more differences. Further, large features also need more computational power. Therefore, considering the large dataset, max_features was set to 2, and *n*_estimators was set to 100 for the RF classifier, which resulted in an accuracy close to that of the model constructed with 500 estimators (0.43–0.81 smaller) (Figure 2).

The Its2vec model was compared with other three predictors in terms of performance. Although the classification accuracy of our model was ~3% lower than that of RDP and Mothur for ITSset_5, it should be noted that the number of features in RDP and Mothur are larger than that in Its2vec. For instance, RDP and Mothur take 8-mer frequency as input, generating 65,536 features, whereas the average number of features used by Its2vec was 700 (the average length of ITS sequences was 693 in this study), which was further reduced to 100 after distributed representation by the word embedding method. Because of this dimensionality reduction, Its2vec can be applied to large databases, while RDP is not suitable for such large datasets because of the computational power requirement. In the Fold-10 dataset, Its2vec showed the best performance. The funbarRF predictor uses *g*-spaced features as input; the number of features of the model was 90 (*g* = 1 + 2 + 3 + 4 + 5), which is close to the number of features generated by word2vec. However, our model achieved significantly higher accuracy than funbarRF in both the ITSset_5 and Fold-10 datasets (Table 5). It should be pointed out that the accuracy might be further improved in several ways, i.e. taking the pseudo components and tertiary structure of the ITS into consideration.

## 5. Conclusion

We presented Its2vec, a bioinformatics tool for the classification of fungal ITS barcodes to the species level. To cover a broad range of fungal taxa, an ITS database covering more than 25,000 species was constructed. For dimensionality reduction, the word embedding algorithm was used to represent the fungal DNA barcode sequences as a dense, 100-dimensional vector. Its2vec achieved an accuracy comparable to those of state-of-the-art predictors. We expect that the Its2vec model will be helpful for the identification of fungal species and, thus, for furthering our understanding of their functional mechanisms and guiding their application in agriculture. Also, computational intelligence such as neural networks [83–85], evolutionary algorithms [86, 87], and unsupervised learning [2, 88, 89] can be applied in this field.

## Figures and Tables

**Table 1:** Taxonomic coverage of the ITS database established in this study.

Taxonomy level	ITS subsets									Number of taxa
	ITSset_2	ITSset_3	ITSset_4	ITSset_5	ITSset_6	ITSset_7	ITSset_8	ITSset_9	ITSset_10	
Phyla	15	13	9	8	7	8	8	7	10	18
Classes	58	46	41	34	31	32	28	30	43	63
Order	165	142	128	106	97	98	78	78	133	187
Family	516	424	381	317	280	263	220	201	418	626
Genus	2073	1432	1116	875	693	633	497	404	1598	3385
Species	8586	4141	2503	1684	1236	929	701	566	5374	25720
Sequences	17172	12423	10012	8420	7416	6503	5608	5094	53740	126388

**Table 2:** Accuracy of the models constructed with different *k*-mers and subsets.

ITSset	3-mer	4-mer	5-mer	6-mer	7-mer	8-mer	9-mer	10-mer	11-mer	12-mer
ITSset_2	70.04	68.08	68.32	69.23	69.64	70.37	71.37	71.23	70.53	69.34
ITSset_3	83.96	82.70	83.53	83.88	83.71	83.94	84.71	84.22	83.41	82.64
ITSset_4	89.15	89.13	89.538	89.63	89.79	89.85	90.40	90.12	89.07	87.95
ITSset_5	92.36	92.71	93.17	93.02	92.84	92.96	93.37	93.15	92.47	91.78
ITSset_6	93.34	93.68	93.99	94.22	93.82	94.05	94.12	94.39	93.31	92.97
ITSset_7	94.99	95.40	95.92	95.73	95.76	96.02	96.03	95.77	94.99	94.88
ITSset_8	96.09	96.20	96.47	96.45	96.34	96.31	96.43	96.36	96.31	95.74
ITSset_9	95.84	96.47	96.54	96.78	96.62	96.58	96.51	96.37	95.90	95.78
ITSset_10	84.37	84.57	85.78	86.37	86.36	87.19	87.96	86.26	86.06	84.93

**Table 3:** Accuracy of the models constructed with different window sizes and subsets.

ITSset	Size = 1	Size = 2	Size = 3	Size = 4	Size = 5	Size = 6	Size = 7
ITSset_2	66.96	70.60	71.19	71.65	71.30	70.75	70.84
ITSset_3	81.26	84.10	84.73	85.20	84.88	84.47	84.15
ITSset_4	87.74	90.26	90.14	90.32	90.39	89.92	89.87
ITSset_5	91.50	93.47	93.69	93.30	93.33	93.10	93.17
ITSset_6	93.03	94.62	94.62	94.30	94.27	94.39	94.30
ITSset_7	95.42	96.28	95.99	95.96	95.85	95.77	95.71
ITSset_8	96.06	97.02	96.63	96.50	96.63	96.67	96.54
ITSset_9	96.43	97.02	96.90	96.84	96.76	96.60	97.02
ITSset_10	85.62	88.00	87.73	87.71	88.06	87.42	96.91

**Table 4:** Performance of the Its2vec model on 9 ITSsets based on optimized parameters.

ITSset	Accuracy	Precision	Recall	MCC
ITSset_2	78.62	72.10	78.62	0.79
ITSset_3	89.70	85.96	89.70	0.90
ITSset_4	93.36	90.69	93.36	0.96
ITSset_5	95.51	93.58	95.51	0.96
ITSset_6	95.95	94.09	95.95	0.96
ITSset_7	96.96	95.62	96.96	0.97
ITSset_8	97.50	96.37	97.50	0.98
ITSset_9	97.53	96.37	97.53	0.98
ITSset_10	90.23	86.48	90.23	0.90

**Table 5:** Comparison of the accuracy of the Its2vec and other existing predictors.

ITS dataset	Classifier	Accuracy	Significance
ITSset_5	Its2vec	95.51 ± 1.55	a^∗^
	RDP	98.68 ± 0.55	b
	Mothur	97.97 ± 0.62	Bc
	funbarRF	91.00 ± 2.656	c

Fold-10	Its2vec	89.80 ± 1.92	a
	RDP	89.36 ± 2.21	a
	Mothur	85.54 ± 2.54	b
	funbarRF	84.94 ± 4.65	b

^∗^Different letters indicate significant differences among the methods according to Tukey's HST test at *P* < 0.05.

## Data Availability

All data and source code were available at http://lab.malab.cn/~wangchao/softwares/software.html.

## Supplementary Materials

Supplementary Materials Supplementary Table S1: precision of the models constructed with different *k*-mers and subsets. Supplementary Table S2: recall of the models constructed with different *k*-mers and subsets. Supplementary Table S3: MCC of the models constructed with different *k*-mers and subsets. Supplementary Table S4: precision of the models constructed with different window sizes and subsets. Supplementary Table S5: recall of the models constructed with different window sizes and subsets. Supplementary Table S6: MCC of the models constructed with different window sizes and subsets.
