# Supplementary material for: Its2vec: Fungal Species Identification Using Sequence Embedding and Random Forest Classification
Source: Biomed Res Int. 2020 May 27;2020:2468789. doi: 10.1155/2020/2468789 (PMC7275950; doi:10.1155/2020/2468789)
Supplement: Supplementary Materials — Supplementary Table S1: precision of the models constructed with different k-mers and subsets. Supplementary Table S2: recall of the models constructed with different k-mers and subsets. Supplementary Table S3: MCC of the models constructed with different k-mers and subsets. Supplementary Table S4: precision of the models constructed with different window sizes and subsets. Supplementary Table S5: recall of the models constructed with different window sizes and subsets. Supplementary Table S6: MCC of the models constructed with different window sizes and subsets. [file 2468789.f1.docx]

**Supplementary Table S1** Precision of the models constructed with different k-mers and subsets

**Supplementary Table S2** Recall of the models constructed with different k-mers and subsets

**Supplementary Table S3** MCC of the models constructed with different k-mers and subsets

**Supplementary Table S4** Precision of the models constructed with different window sizes and subsets

**Supplementary Table S5** Recall of the models constructed with different window sizes and subsets

**Supplementary Table S6** MCC of the models constructed with different window sizes and subsets
